# Detection of Extended Spectrum Beta-Lactamases Resistance Genes among Bacteria Isolated from Selected Drinking Water Distribution Channels in Southwestern Nigeria

**DOI:** 10.1155/2016/7149295

**Published:** 2016-08-03

**Authors:** Ayodele T. Adesoji, Adeniyi A. Ogunjobi

**Affiliations:** ^1^Department of Biological Sciences, Federal University Dutsin-Ma, Katsina Road, PMB 5001, Dutsin-Ma, Katsina State, Nigeria; ^2^Department of Microbiology, University of Ibadan, Oyo State, Nigeria

## Abstract

Extended Spectrum Beta-Lactamases (ESBL) provide high level resistance to beta-lactam antibiotics among bacteria. In this study, previously described multidrug resistant bacteria from raw, treated, and municipal taps of DWDS from selected dams in southwestern Nigeria were assessed for the presence of ESBL resistance genes which include *bla*_TEM_, *bla*_SHV_, and *bla*_CTX_ by PCR amplification. A total of 164 bacteria spread across treated (33), raw (66), and municipal taps (68), belonging to *α*-Proteobacteria, *β*-Proteobacteria, γ-Proteobacteria, Flavobacteriia, Bacilli, and Actinobacteria group, were selected for this study. Among these bacteria, the most commonly observed resistance was for ampicillin and amoxicillin/clavulanic acid (61 isolates). Sixty-one isolates carried at least one of the targeted ESBL genes with *bla*_TEM_ being the most abundant (50/61) and *bla*_CTX_ being detected least (3/61).* Klebsiella* was the most frequently identified genus (18.03%) to harbour ESBL gene followed by* Proteus* (14.75%). Moreover, combinations of two ESBL genes, *bla*_SHV_ + *bla*_TEM_ or *bla*_CTX_ + *bla*_TEM_, were observed in 11 and 1 isolate, respectively. In conclusion, classic *bla*_TEM_ ESBL gene was present in multiple bacterial strains that were isolated from DWDS sources in Nigeria. These environments may serve as foci exchange of genetic traits in a diversity of Gram-negative bacteria.

## 1. Introduction

Access to safe drinking water is essential for human health [1]. While access to safe and affordable water should be available to everyone, this remains a challenge in low- and middle-income countries including Nigeria, which is the most populous country in Africa. Safe drinking water is mostly viewed in terms of organic and inorganic contaminants, but also in terms of biological contamination. In this respect, less attention has been given to the role that water may play in the dissemination of antibiotic resistance traits in populations that are exposed to substandard water on a daily basis [2–5].

Arguably, the most clinically important antibiotic resistance genes are those that encode enzymes that hydrolyze *β*-lactams (*bla* genes) [6]. These traits confer high level resistance to *β*-lactam antibiotics, which are the most widely used antibiotics in clinical and veterinary practice [7, 8]. Extended Spectrum *β*-Lactamase (ESBL) is group of enzymes that can hydrolyze a variety of *β*-lactams including cephalosporins like ceftazidime, cefotaxime, and ceftriaxone and monobactams like aztreonam in addition to penicillin but does not hydrolyze cephamycins like cefoxitin. Most of the ESBL also have the ability to hydrolyze fourth-generation cephalosporins including cefepime [9].

A variety of transferable genes encoding *β*-lactamase activity have been described in clinical environments including *bla*_CTX-M_, *bla*_GES_, *bla*_HER_, *bla*_OXA_, *bla*_OXY_, *bla*_SED_, *bla*_SHV_, *bla*_SPM_, *bla*_VEB_, *bla*_VIM_, and* ampC *[10]. Among the most common* bla* genes is the *bla*_TEM-1_ gene, the first described* bla* gene and a representative of the *bla*_TEM_ group that now consists of more than 220 different distinct variants (“alleles”), which encode different amino acid polymorphisms that extend their substrate range (http://www.lahey.org/Studies/temtable.asp) [10].

Previous reports indicated that multidrug resistant bacteria are present in drinking water distribution systems from southwestern Nigeria [3–5]. These bacteria encoded resistance to a diversity of beta-lactams including ceftiofur, ampicillin, and combination of amoxicillin and amoxicillin/clavulanic acid.

The aim of this study was to genotype MDR bacteria isolated from our previous studies [3–5] for the presence of selected beta-lactamase resistance genes using PCR.

## 2. Materials and Methods

### 2.1. Dam Description, Sampling, Selection, Isolation, Storage, and Molecular Characterization of Bacteria

The description of sampled dams in this study is in our previous publications [3–5]. Moreover, for clarity of this paper, ninety-six water samples were purposively collected aseptically into sterile screw cap bottles from six selected water distribution systems of dams in Ife, Ede, Asejire, Eleyele, Owena Ondo, and Owena-Idanre in southwestern Nigeria. Samples were collected four times between December 2010 and July 2011 from raw, treated, and two randomly selected municipal distribution taps. Afterwards, samples were serially diluted and plated on Nutrient agar, eosin methylene blue agar (EMB), and Deoxycholate agar (DCA). Thereafter, bacteria were picked with the aim of maximizing the diversity of colony morphology represented from each sample. Picked colonies were restreaked on Nutrient agar to obtain pure cultures. These were subsequently transferred to Nutrient agar slants and also stored in phosphate buffer glycerol at −80°C [3–5]. Molecular characterization of bacteria using 16S rDNA sequencing was determined as described in Adesoji et al. [11].

### 2.2. Antibiotic Susceptibility Testing

Agar dilution assays (also called breakpoint assays) were conducted using Luria-Bertani agar with seeded antibiotics used to assess antibiotic susceptibility. Antibiotics concentrations used for Gram-negative bacteria included florfenicol (16 *μ*g/mL), tetracycline (16 *μ*g/mL), streptomycin (16 *μ*g/mL), gentamycin (16 *μ*g/mL), kanamycin (64 *μ*g/mL), chloramphenicol (32 *μ*g/mL), nalidixic acid (30 *μ*g/mL), amoxicillin/clavulanic acid (32/16 *μ*g/mL), ceftiofur (12 *μ*g/mL), sulfamethoxazole (512 *μ*g/mL), and sulfamethoxazole/trimethoprim (76/4 *μ*g/mL). Antibiotics concentrations used for Gram-positive bacteria include sulfamethoxazole (512 *μ*g/mL), ampicillin (0.5 *μ*g/mL), tetracycline (16 *μ*g/mL), sulfamethoxazole/trimethoprim (76/4 *μ*g/mL), gentamycin (16 *μ*g/mL), erythromycin (8 *μ*g/mL), rifampin (4 *μ*g/mL), lincomycin (4 *μ*g/mL), and ciprofloxacin (4 *μ*g/mL). Negative and positive controls used were* E. coli* strain K12 and* E. coli* strain H4H, respectively, as we described in our previous studies [3–5, 11].

### 2.3. Resistance Genotyping

PCR testing was conducted for bacteria having resistance to ≥3 classes of antibiotics including resistance to amoxicillin/clavulanic acid, ceftiofur, or ampicillin. Thereafter, forward and reverse primer specific for selected ESBL genes included *bla*_SHV_ (SHV_F, 5′-GCGAAAGCCAGCTGTCGGGC-3′ and SHV_R, 5′-GATTGGCGGCGCTGTTATCGC-3), *bla*_CTX_M_ (CTX_F, 5′-GTGCAGTACCAGTAAAGTTATGG-3′ and CTX_R, 5′-CGCAATATCATTGGTGGTGCC-3′), and *bla*_TEM_ (TEM_F, 5′-AAAGATGCTGAAGATCA-3′ and TEM_R, 5′-TTTGGTATGGCTTCATTC-3′) [12]. Condition for *bla*_SHV_ PCR included 1 min denaturation (95°C followed by 30 cycles of 96°C for 30 s, 62°C for 30 s, and 72°C for 30 s and final extension of 72°C for 10 min. Conditions were identical for other assays except the annealing temperatures which were 55°C and 44°C for *bla*_CTX-M_ and *bla*_TEM_, respectively. Afterwards, PCR products were separated, sized, and visualized by using 1% agarose gel electrophoresis to confirm amplification.

## 3. Results

### 3.1. Bacteria Isolates

Isolates used in this study were selected from our previous studies [3–5] and represented *α*-Proteobacteria, *β*-Proteobacteria, *γ*-Proteobacteria, Flavobacteriia, Bacilli, and Actinobacteria with 33, 66, and 68 being isolated from all treated, raw, and municipal taps, respectively (Table 1).* Proteus* was the most frequent (18.18%) isolated Gram-negative genus from the treated water while* Klebsiella* was the most frequently (15.15%) isolated genus from raw water.* Bacillus* was the most common isolated Gram-positive genus for treated and municipal water.

### 3.2. PCR-Positive Isolates

In this study, 61 isolates out of 164 MDR isolates were PCR-positive for at least one targeted gene. Highest occurrence of* bla* gene among Gram-negative bacteria compared to Gram-positive bacteria was observed. Most commonly isolated genus carrying* bla* gene is* Klebsiella* (18.03%) followed by* Proteus* spp. (14.75%). *Bla*_TEM_ was detected in the majority of beta-lactam resistant isolates (50/61) while *bla*_CTX_ was rarely detected (3/61) (Table 2). A combination of two genes, *bla*_SHV_ + *bla*_TEM_ or *bla*_CTX_ + *bla*_TEM_, was observed in 11 and 1 bacteria, respectively (Table 2). Other genera, including* Aquitalea*,* Comamonas*,* Enterobacter*,* Leucobacter*,* Lysinibacillus*,* Pantoea*,* Pseudochrobactrum*,* Sphingobacterium*, and* Ralstonia*, were tested but were PCR-negative for resistance genes.

## 4. Discussion

The hazard associated with the pathogenicity of microbes is aggravated by its ability to resist destruction by antibiotics [2]. In this study, beta-lactamase producing bacteria and genes (i.e., *bla*_CTX_ and *bla*_TEM_) were detected from every sampled water distribution system. This is similar to the report of Xi et al. [13] who also investigated the prevalence and dynamics of heterotrophic antibiotics resistance bacteria and genes in drinking water source and treated drinking water using culture-dependent methods and molecular techniques. The authors observed the presence of *bla*_TEM_ and *bla*_SHV_ genes in all water samples except one, which is evidence that these genes are distributed widely in drinking water systems. This is similar to what we also reported in Table 3, showing the spread of these beta-lactamase resistance genes among all raw, final, and municipal tap sources. The results showed that even among bacteria from the municipal tap and final treated water from the dam which are the point of consumer consumption *bla*_TEM_ and *bla*_CTX_ occurred among bacteria from these sources in high number. Xi et al. [13] also observed selective increases in the levels of both genes in tap water due to either water treatment or regrowth within drinking water distribution systems. This, as they therefore reported, suggested the spread of at least some beta-lactam-resistant determinants through drinking water distribution systems. However, in this study, it is important to point out that every site is different for numerous variables making it impossible to derive meaningful correlations between water treatment practices and the occurrence of beta-lactamase resistance genes. Given these differences, the only practical means to assess the effects of water treatment practices (which is not our goal with this paper) would be to test changes with experimental manipulation. We have been reluctant to provide significant detail of the sample sites precisely because we do not wish to imply that there are defendable correlations between occurrence and site characteristics, nor do we wish to encourage readers to draw such inferences.

Moreover, we observed that there were beta-lactam resistant strains that were negative for the PCR assays used in this study. The most commonly detected* bla* genes were *bla*_TEM_ and *bla*_SHV_ among* Klebsiella*. This finding is contrary to previous reports [14–16]. They observed dominance of *bla*_CTX_ among non-TEM and SHV bacteria from clinical environment which were similar to what Ojdana et al. [17] reported among clinical samples from Poland. Additionally, studies on* Pseudomonas* spp. isolated from these water distribution systems also observed a higher occurrence of *bla*_TEM_ (40.9%) and *bla*_CTX_ (27.3%) while none of the pseudomonads showed the presence of *bla*_CTX_ [18]. Our observation of the highest occurrence of* bla* gene among Gram-negative bacteria, when compared to Gram-positive bacteria, in this study is similar to reports of [19, 20]. These authors also confirm *bla*_TEM_ that was frequently detected among Gram-negative bacteria from this study as the most common* bla* gene in their studies.

In this study, environmental bacteria belonging to each of these genera* Bordetella*,* Brevundimonas*,* Chromobacterium*,* Providencia*,* Psychrobacter*,* Stenotrophomonas*,* Trabulsiella*, and* Aeromonas* possess at least one of the beta-lactamase resistance genes tested; the most common among them is *bla*_TEM_. Occurrence of this gene in these environmental isolates is contrary to the report that ESBL production is mostly found to occur among enteric species [21]. The first* bla* genes (*bla*_BOR-1_ and *bla*_OXA-2_) were reported in* Bordetella* by Kadlec et al. [22]. However, we did not come across any publication where *bla*_TEM_ has been reported in this bacterium. This could be the first report of this gene in this bacterium. Nevertheless, *bla*_TEM_ has been reported in* Providencia* [23],* Stenotrophomonas* [24], and* Aeromonas* [25]. In fact, another* bla* gene such as *bla*_LI_ has been reported in* Stenotrophomonas* from China [26] and *bla*_SHV_ and *bla*_CTX-M_ have been reported in* Aeromonas* [25]. Moreover, no *bla*_SHV_ has also been reported in* Trabulsiella*; this report probably might be its first description.

The association of more than one *β*-lactamase within the same isolate has been reported [27, 28]. However, from our studies the most common of this association is *bla*_SHV_ + *bla*_TEM_. This was detected among* Acinetobacter*,* Alcaligenes*,* Bacillus*,* Klebsiella,* and* Stenotrophomonas* while the combination of *bla*_CTX_ and *bla*_TEM_ was only observed in* Klebsiella*. This occurrence denotes the wider dissemination of these* bla* genes probably due to involvement of genetic element in mobilization of these genes [29]. These same authors [29] also observed various combinations of *bla*_CTX_, *bla*_TEM_, and *bla*_SHV_ in* Klebsiella* from clinical isolates in India.

The occurrence of ESBL genes among bacteria from this study has a public health implication. Previous studies have shown that potential ESBL species such as* K. pneumonia*, the most frequent bacterium with* bla* gene in this study, and* E. coli* have a high tendency to possess and transfer* bla* genes [30]. However, this may occur through conjugation because the genes are often found in mobile elements like transposons and integrons [31]. Some of these species may be pathogenic strains that have the potential to cause life-threatening diseases and widespread outbreak. For instance, Zhang et al. [32] have reported that *bla*_CTX-M_ and *bla*_TEM_ genes in opportunistically pathogenic* Klebsiella* spp. have been associated with nosocomial infections and outbreak of diarrhea. Therefore, occurrence of these bacteria especially in the drinking water poses a lot of danger to health, economy, and social well-being of consumers. This populace could also be exposed to these genes carrying pathogenic species in food and food products by the use of the contaminated water for domestic purposes, farming, and agriculture. It should also be noted that the fact that these species are multidrug resistance deepens the gravity of the situation. However, from our observation during sampling, the possible source of these MDR bacteria especially in the raw water could be from run-off from agricultural farmlands located very close to some of these constructed dams [4]. Some of these farmlands make use of organic fertilizers which may consist of unmetabolized antibiotics which may eventually get to the water through run-off, which may cause selective pressure on the bacteria in the aquatic systems.

From the bacteria found in Nigeria, many studies have described the occurrence of* bla* genes among clinical isolates. For example, Akujobi et al. [33] reported *bla*_TEM_ in* E. coli* while Akinniyi et al. [34] reported the gene not only in* E. coli* but also among* Klebsiella*,* Salmonella*,* Citrobacter*,* Enterobacter*,* Pseudomonas,* and* Proteus.* The highest prevalence (5.6%) was also in* Klebsiella* which is similar to our findings. However, from environmental isolates few studies seem to have been conducted. Moreover, Adelowo et al. [35] reported *bla*_TEM_ in* E. coli* from well water while Chikwendu et al. [36] described not only *bla*_TEM_ among* Pseudomonas* from river and aquaculture samples but also *bla*_SHV_. Moreover, this study seems to be the first report describing these genes among a wide diversity of environmental bacteria from Nigeria drinking water distribution systems. It is therefore important to raise public and health worker awareness in terms of prevention of outbreak of MDR infectious pathogens among consumers. This also undermines the need for government agencies controlling these dams and health organizations to initiate measures to effectively control the release of contaminant into the environment. It would also be good to continually isolate bacteria from other water distribution systems in Nigeria and to carry out further molecular testing for the presence of other* bla *genes, characterize, and determine whether these genes are present on transferable plasmids, transposons, or integrons, which would enhance easy spreading.

## 5. Conclusion

The occurrence of beta-lactamase producing bacteria and genes in all sampled water in this study, especially treated drinking water, showed that these water distribution systems could serve as a vehicle for transmission of these antibiotic resistance bacteria and genes to consumers, hence, of a great public health concern.

## Figures and Tables

**Table 1:** Classes and families of selected bacteria.

Source	Class	Family	Number (% of total from source)
Raw water	*α*-Proteobacteria	Brucellaceae	1 (1.51)
	*β*-Proteobacteria	Alcaligenaceae	9 (13.64)
		Neisseriaceae	1 (1.51)
	*γ*-Proteobacteria	Enterobacteriaceae	27 (40.91)
		Moraxellaceae	2 (3.03)
		Aeromonadaceae	6 (9.09)
		Xanthomonadaceae	2 (3.03)
	Flavobacteriia	Myroidaceae	1 (1.51)
	Uncultured bacteria clone		3 (4.55)
	Bacilli	Bacillaceae	10 (15.15)
		Staphylococcaceae	3 (4.55)
	Actinobacteria	Microbacteriaceae	1 (1.51)
	*Total raw water*		*66*

Treated water	*α*-Proteobacteria	Caulobacteraceae	1 (3.03)
	*β*-Proteobacteria	Alcaligenaceae	5 (15.15)
		Neisseriaceae	1 (3.03)
	*γ*-Proteobacteria	Enterobacteriaceae	10 (30.30)
	Flavobacteriia	Myroidaceae	1 (3.03)
	Uncultured bacteria clone		2 (6.06)
	Bacilli	Bacillaceae	12 (36.36)
		Staphylococcaceae	1 (3.03)
	*Total treated water*		*33*

Municipal taps	*α*-Proteobacteria	Caulobacteraceae	1 (1.47)
	*β*-Proteobacteria	Alcaligenaceae	7 (10.29)
		Neisseriaceae	3 (4.41)
	*γ*-Proteobacteria	Enterobacteriaceae	21 (30.88)
		Moraxellaceae	6 (8.82)
	Flavobacteriia	Myroidaceae	3 (4.41)
	Uncultured bacteria clone		
	Bacilli	Bacillaceae	26 (38.24)
		Staphylococcaceae	1 (1.47)
	*Total municipal tap*		*68*

Note: identification was based on 16S rDNA sequencing. These bacteria were obtained from our previous works [3–5].

**Table 2:** Characterization of a number of cultured bacterial isolates encoding different *ESBL *genotypes.

Genus/species/accession number	Source	Resistant phenotypes	*bla*_TEM_	*bla*_SHV_	*bla*_CTX_
*Dam 1* ^a^					
*Escherichia coli* AP010960.1	DAM 1 IRW	T, AM, S, C, N, SXT, SU	*bla*_TEM_		
Uncultured bacterium clone JN595783.1	DAM 1 IFFW	T, FF, AM, G, SU	*bla*_TEM_		
*Bacillus thuringiensis* JN377782.1	DAM 1 IFFW	SU, AM, T, E, SXT, RIF, LIN, GEN	*bla*_TEM_		
*Brevundimonas diminuta* EU545397.1	DAM 1 IFFW	S, G, K, N, AM, SXT, SU		*bla*_SHV_	
*Proteus mirabilis* AB626123.1	DAM 1 IFFW	FF, T, S, G, K, C, AMC, AM, SU, SXT	*bla*_TEM_		
*Bacillus thuringiensis* JN377782.1	DAM 1 IFM1	SU, AM, E, SXT, RIF, LIN	*bla*_TEM_	*bla*_SHV_	

*Dam 2* ^b^					
*Bacillus altitudinis* HQ432811.1	DAM 2 EDRW	SU, E, RIF, LIN, AM		*bla*_SHV_	
*Bordetella *sp. HQ840720.1	DAM 2 EDRW	T, FF, S, C, N, CEF, AM, SXT, SU	*bla*_TEM_		
*Proteus vulgaris* JN630888.1	DAM 2 EDRW	T, AM, SXT, SU	*bla*_TEM_		
*Staphylococcus *sp. JN695710.1	DAM 2 EDRW	SU, T, E, SXT, RIF, LIN, AM	*bla*_TEM_		
*Stenotrophomonas maltophilia* JN703732.1	DAM 2 EDRW	T, S, K, CEF, AM, AMC, SU	*bla*_TEM_	*bla*_SHV_	
*Bacillus cereus* AP007209.1	DAM 2 EDFW	SU, AM, T, E, SXT, RIF, LIN	*bla*_TEM_		
*Morganella *sp. GQ179706.1	DAM 2 EDFW	T, S, AM, SXT, SU	*bla*_TEM_		
*Psychrobacter *sp. HQ730697.1	DAM 2 EDM2	T, S, CEF, AM, SXT, SU	*bla*_TEM_		

*Dam 3* ^c^					
*Alcaligenes faecalis* JN162124.1	DAM 3 ARW	S, CEF, AM, SXT, SU		*bla*_SHV_	
*Klebsiella pneumoniae* AB675600.1	DAM 3 ARW	FF, T, S, C, AMC, CEF, AM, SU, SXT	*bla*_TEM_		
*Leucobacter komagatae* AJ746337.1	DAM 3 ARW	T, S, AM, G, K, SXT, N, SU	*bla*_TEM_		
*Proteus mirabilis* AB626123.1	DAM 3 ARW	T, S, AM, N, SXT, SU	*bla*_TEM_		
Uncultured bacterium clone JN595783.1	DAM 3 ARW	T, G, K, C, N, CEF, AM, SXT, AMC, SU	*bla*_TEM_		
*Bacillus pumilus* EF010673.1	DAM 3 AFW	SU, AM, T, E, SXT, RIF, LIN	*bla*_TEM_		
*Klebsiella pneumoniae* JF919909.1	DAM 3 AFW	T, S, C, AM, SXT, SU		*bla*_SHV_	
*Myroides odoratus* AB517709.1	DAM 3 AFW	FF, T, S, G, K, C, AM, SXT, AMC, SU	*bla*_TEM_		
*Proteus vulgaris* JN630888.1	DAM 3 AFW	FF, T, S, C, N, CEF, AM, SXT, AMC, SU	*bla*_TEM_		
*Acinetobacter calcoaceticus*	DAM 3 AM2	S, AMC, AM, SU	*bla*_TEM_	*bla*_SHV_	
*Chromobacterium *sp. AB426118.1	DAM 3 AM1	T, S, CEF, AM, SXT, SU, AMC, SU	*bla*_TEM_		
*Klebsiella pneumoniae* JF513171.1	DAM 3 AM2	FF, C, CEF, AM, SXT, AMC, SU, AMC, SU	*bla*_TEM_	*bla*_SHV_	

*Dam 4* ^d^					
*Aeromonas caviae* AB626132.1	DAM 4 ERW	T, S, AM, SXT, N, AMC, SU	*bla*_TEM_		
*Alcaligenes faecalis* HQ161777.1	DAM 4 ERW	T, S, K, AM, SU	*bla*_TEM_		
*Alcaligenes faecalis* JN162124.1	DAM 4 ERW	T, S, K, AM, SU	*bla*_TEM_		
*Klebsiella pneumoniae* JN545039.1	DAM 4 ERW	S, CEF, AM, SXT, AMC, SU	*bla*_TEM_		
*Klebsiella pneumoniae* JN545039.1	DAM 4 ERW	T, K, N, AM, SU	*bla*_TEM_	*bla*_SHV_	
*Klebsiella pneumoniae* JF513172.1	DAM 4 ERW	T, FF, S, C, AM, SXT, AMC, SU	*bla*_TEM_	*bla*_SHV_	
*Morganella morganii* FJ971868.1	DAM 4 ERW	T, S, K, CEF, AM, SXT, AMC, SU	*bla*_TEM_		
*Proteus vulgaris* JN630888.1	DAM 4 ERW	T, C, CEF, AM	*bla*_TEM_		
*Proteus mirabilis* GU420988.1	DAM 4 ERW	T, S, K, N, AM, SXT, AMC, SU	*bla*_TEM_		
*Proteus vulgaris* JN630888.1	DAM 4 ERW	FF, T, S, G, K, C, AM, SXT, N, AMC, SU	*bla*_TEM_		
*Providencia vermicola* NR_042415.1	DAM 4 ERW	T, G, AM, SU	*bla*_TEM_		
*Trabulsiella guamensis* AB273737.1	DAM 4 ERW	T, C, CEF, AM, SU, SXT		*bla*_SHV_	

*Dam 5* ^e^					
*Klebsiella *sp. JN036433.1	DAM 5 OWODFW	T, FF, S, C, AM, SXT, AMC, SU	*bla*_TEM_	*bla*_SHV_	
*Alcaligenes faecalis* JN162124.1	DAM 5 OWODM2	T, S, G, K, C, AM, SXT, SU	*bla*_TEM_		
*Escherichia coli* CP003034.1	DAM 5 OWODM2	T, AM, AMC, SU	*bla*_TEM_		
*Escherichia coli* CP003034.1	DAM 5 OWODM2	T, AM, AMC, SU	*bla*_TEM_		
*Morganella morganii* AM931264.1	DAM 5 OWODM1	T, S, CEF, AM, SXT, AMC, SU	*bla*_TEM_		
*Morganella morganii* AB089245.1	DAM 5 OWODM3	T, S, K, CEF, SXT, AMC, SU	*bla*_TEM_		
*Myroides odoratus* AB517709.1	DAM 5 OWODM3	T, S, G, K, CEF, AM, SXT, AMC, SU	*bla*_TEM_		
*Serratia marcescens* FJ607982.1	DAM 5 OWODM3	T, AM, AMC, CEF, SU		*bla*_SHV_	

*Dam 6* ^f^					
*Acinetobacter baumannii* JF919866.1	DAM 6 OWIRW	T, FF, S, C, AM, SXT, AMC, SU	*bla*_TEM_		
*Bacillus thuringiensis* JN377782.1	DAM 6 OWIRW	SU, AM, T, SXT, RIF, LIN, CIP, GEN	*bla*_TEM_	*bla*_SHV_	
*Klebsiella *sp. DQ989215.2	DAM 6 OWIRW	T, S, AM, SXT, SU	*bla*_TEM_		*bla*_CTX_
*Klebsiella pneumoniae* CP002910.1	DAM 6 OWIRW	T, S, C, AM, SXT, AMC, SU	*bla*_TEM_	*bla*_SHV_	
*Klebsiella oxytoca* JF317350.1	DAM 6 OWIRW	K, AM, SXT, SU			*bla*_CTX_
*Morganella morganii* AB089245.1	DAM 6 OWIRW	T, S, AM, AMC, SU			*bla*_CTX_
*Proteus vulgaris* JN630888.1	DAM 6 OWIRW	T, FF, S, C, CEF, SXT, N, AMC, SU	*bla*_TEM_		
*Serratia marcescens* JF429936.1	DAM 6 OWIRW	T, S, C, CEF, AM, AMC, SU		*bla*_SHV_	
Uncultured bacterium JN595068.1	DAM 6 OWIRW	S, G, K, AM, SU	*bla*_TEM_		
*Bacillus altitudinis* HQ432811.1	DAM 6 OWIFW	SU, AM, T, E, SXT, RIF, LIN	*bla*_TEM_	*bla*_SHV_	
*Alcaligenes *sp. JF707602.1	DAM 6 OWIM1	T, S, G, K, CEF, AM, AMC, SU	*bla*_TEM_		
*Alcaligenes faecalis* HM145896.1	DAM 6 OWIM1	T, S, G, K, CEF, AM, AMC, SU	*bla*_TEM_	*bla*_SHV_	
*Alcaligenes *sp. JF303893.1	DAM 6 OWIM2	T, S, G, K, N, CEF, AM, SU		*bla*_SHV_	
*Citrobacter freundii* JN644567.1	DAM 6 OWIM1	T, S, AM, SXT, N, AMC, SU	*bla*_TEM_		
*Klebsiella pneumoniae* CP002910.1	DAM 6 OWIM1	T, S, CEF, AM, SU		*bla*_SHV_	
*Proteus mirabilis* AB626123.1	DAM 6 OWIM2	T, S, C, N, AMC, SXT		*bla*_SHV_	

*Codes*. ^a^Obafemi Awolowo University, Ife, Osun State; IRW = Ife raw water; IFFW = Ife treated water; IFM1 = Ife municipal tap 1; IFM2 = Ife municipal tap 2; ^b^Ede, Osun State; EDRW = Ede raw water; EDFW = Ede treated water; EDM1 = Ede municipal tap 1; EDM2 = Ede municipal tap 2; ^c^Asejire, Oyo State, Nigeria; ARW = Asejire raw water; AFW = Asejire treated water; AM1 = Asejire municipal tap 1; AM2 = Asejire municipal tap 2; ^d^Eleyele, Oyo State; ERW = Eleyele raw water; EFW = Eleyele treated water; EM1 = Eleyele municipal tap 1; EM2 = Eleyele municipal tap 2; ^e^Owena Ondo, Ondo State; OWODRW = Owena Ondo raw water; OWODFW = Owena Ondo treated water; OWODM1 = Owena ondo municipal tap 1; OWODM2 = Owena Ondo municipal tap 2; ^f^Owena-Idanre, Ondo State; OWIRW = Owena-Idanre raw water; OWIFW = Owena-Idanre treated water; OWIM1 = Owena-Idanre municipal tap 1; OWIM2 = Owena-Idanre municipal tap 2. Note: bacteria was identified to the genus level by 16S rDNA partial sequence.

Ampicillin (AM); ceftiofur (CEF); chloramphenicol (C); florfenicol (FF); kanamycin (K); streptomycin (S); gentamycin (GEN); tetracycline (T); nalidixic acid (N); sulfamethoxazole (SU); sulfamethoxazole/trimethoprim (SXT); amoxicillin/clavulanic acid (AMC); erythromycin (E); rifampin (RIF); lincomycin (LIN); ciprofloxacin (CIP).

**Table 3:** Number of bacteria and genes observed from all raw, treated, and municipal taps carrying at least one *bla* gene tested for in this study.

Genus	*bla* genes from raw water				*bla* genes from final water				*bla* genes from municipal taps			
	Bacteria number	*bla*_TEM_	*bla*_SHV_	*bla*_CTX_	Bacteria number	*bla*_TEM_	*bla*_SHV_	*bla*_CTX_	Bacteria number	*bla*_TEM_	*bla*_SHV_	*bla*_CTX_
*Acinetobacter* spp.	1	1	0	0	0	0	0	0	1	1	1	0
*Aeromonas* spp.	1	1	0	0	0	0	0	0	0	0	0	0
*Alcaligenes* spp.	3	2	1	0	0	0	0	0	4	3	1	0
*Bacillus* spp.	2	1	2	0	4	4	1	0	1	1	1	0
*Bordetella* spp.	1	1	0	0	0	0	0	0	0	0	0	0
*Brevundimonas* spp.	0	0	0	0	1	0	1	0	0	0	0	0
*Chromobacterium *spp.	0	0	0	0	0	0	0	0	1	1	0	0
*Citrobacter *spp.	0	0	0	0	0	0	0	0	1	1	0	0
*E. coli *	1	1	0	0	0	0	0	0	2	2	0	0
*Klebsiella* spp.	7	6	3	1	2	0	1	0	2	1	2	0
*Leucobacter* spp.	1	1	0	0	0	0	0	0	0	0	0	0
*Morganella* spp.	2	1	0	1	1	1	0	0	2	2	0	0
*Myroides* spp.	0	0	0	0	1	1	0	0	1	1	0	0
*Proteus* spp.	6	6	0	0	2	2	0	0	1	0	1	0
*Providencia* spp.	1	1	0	0	0	0	0	0	0	0	0	0
*Psychrobacter *spp.	0	0	0	0	0	0	0	0	1	1	0	0
*Serratia* spp.	1	0	1	0	0	0	0	0	1	0	1	0
*Staphylococcus* spp.	1	1	0	0	0	0	0	0	0	0	0	0
*Stenotrophomonas* spp.	1	1	1	0	0	0	0	0	0	0	0	0
*Trabulsiella* spp.	1	0	1	0	0	0	0	0	0	0	0	0
Uncultured bacteria clone	2	2	0	0	1	1	0	0	0	0	0	0

*Total*	*32*	*26*	*9*	*2*	*12*	*9*	*3*	*0*	*18*	*14*	*7*	*0*
